# Effect of multiple calcination cycles on CO_2_ capture efficiency during carbonation of MgO in a mineral looping process

**DOI:** 10.1038/s41598-025-23708-2

**Published:** 2025-11-10

**Authors:** Elena Tajuelo Rodriguez, Lawrence M. Anovitz, Sai Adapa, Ke Yuan, Dale Hensley, Dong Youn Chung, Matthew G. Boebinger, Andrew G. Stack, Juliane Weber

**Affiliations:** 1 Nuclear Energy and Fuel Cycle Division, Oak Ridge National Laboratory, Oak Ridge, 37831 USA; 2 Chemical Science Division, Oak Ridge National Laboratory, Oak Ridge, 37831 USA; 3 Center for Nanophase Materials Sciences, Oak Ridge National Laboratory, Oak Ridge, 37831 USA; 4 Department of Geosciences, The Pennsylvania State University, State College, PA 16802 USA

**Keywords:** Mineral looping, Direct air capture, Magnesium oxide, Ambient weathering, Calcination, Environmental chemistry, Carbon capture and storage

## Abstract

Magnesium oxide (MgO) is considered as a potential sorbent for direct air capture of carbon dioxide in a looping process. Previous research on mineral looping for carbon capture from flue gas using MgO has shown deactivation of the sorbent with repeated cycles but repeated cycling for carbonation at ambient conditions has not been yet investigated. Here, we tested three cycles of carbonation for MgO nano-scale powders with different starting surface area. We find that carbonation efficiency is higher at higher surface area. No decrease in carbonation efficiency was observed with cycling, instead we find that carbonation efficiency and surface area are stable or even increasing with cycling. Based on our experimental data, we hypothesize that the carbonation in presence of relative humidity leads to first hydration of MgO, leading to formation of brucite (Mg(OH)_2_). This formation of Mg(OH)_2_ is a volume-increasing reaction, which leads to fracturing of MgO particles and results in an increase in surface area. We observed formation of amorphous and crystalline hydrated carbonates. The crystalline phase observed was nesquehonite (MgCO_3_ ⋅ 3H_2_O). Our results show that MgO does not show any deactivation with repeated cycling for carbon capture at ambient conditions and in the presence of humidity. These findings therefore indicate that MgO is a suitable candidate as sorbent material for direct air capture of carbon dioxide.

**Supplementary Information:**

The online version contains supplementary material available at 10.1038/s41598-025-23708-2.

## Introduction

Previous studies have suggested that alkaline metal oxides may be used for economic direct air capture of CO_2_ by mineral looping^1,2^. A techno-economic analysis of mineral looping using MgO proposed this method as an inexpensive pathway that could scale to gigatons of CO_2_^2^. Currently, calcium oxide (CaO) is also considered the most viable starting material for mineral looping,^3^ which has the benefit of a fast carbonation rate (hours)^3^ but requires a high calcination temperature (900 °C). The alternative material is MgO, which carbonates more slowly, but benefits from a lower calcination temperature (300–500 °C) that requires less energy input^4–6^. The process is envisioned as a thin layer of MgO powder which reacts with the surrounding air for a year^2^ (shorter time scales are being used for CaO^7)^. MgO could be either sourced from calcination of magnesite^8^ or via precipitation of Mg(OH)_2_ from brines or seawater^9^.

One cost in this method is the capital costs of procuring the source material, which is directly linked to the carbonation efficiency or amount of CO_2_ that can be absorbed. A potentially significant issue is that previous research on using mineral looping by MgO or CaO for carbon capture from flue gas or combustion processes, i.e. at elevated temperatures, has indicated that both materials deactivate with repeated cycles,^10^ i.e., contain a reduced reactivity towards carbonation with increasing number of cycles. For example, a 30–45% deactivation with 20 cycles has been reported for both CaO and MgO^10^. The deactivation is attributed to a loss in surface area,^11–14^ presumably due to Ostwald ripening or sintering under the elevated temperatures present during reaction or during calcining, and is a separate issue from passivation during carbonation^3,15^. There are some studies that suggest that MgO may suffer less from surface area loss, e.g., 5–7% loss after 10 cycles^16,17^, but these have not been substantiated (and surface area loss may be temperature dependent). However, since existing studies have focused on capturing CO_2_ from flue gas and thus experiments have been performed at elevated temperature and pressure, there is a lack of knowledge about if deactivation with repeated cycling affects MgO-based mineral looping for direct air capture occurring under ambient temperatures and pressures. Thus here, we report on tests for whether carbonation efficiency changes with repeated cycling under ambient pressures and temperature more relevant to direct air capture. We cycled three MgO powders through carbonation and calcining. In order to eliminate CO_2_ concentration as a limiting variable affecting passivation extent, the experiments were performed in excess CO_2_, specifically 1 atm. Carbonation efficiency was assessed, and carbonation products were determined, and surface area changes were monitored after each calcination cycle. Three cycles of carbonation and calcination were performed since prior work has demonstrated that the largest changes occur in these initial cycles^10,18^.

## Results

### Carbonation efficiency and products

**Fig. 1:**
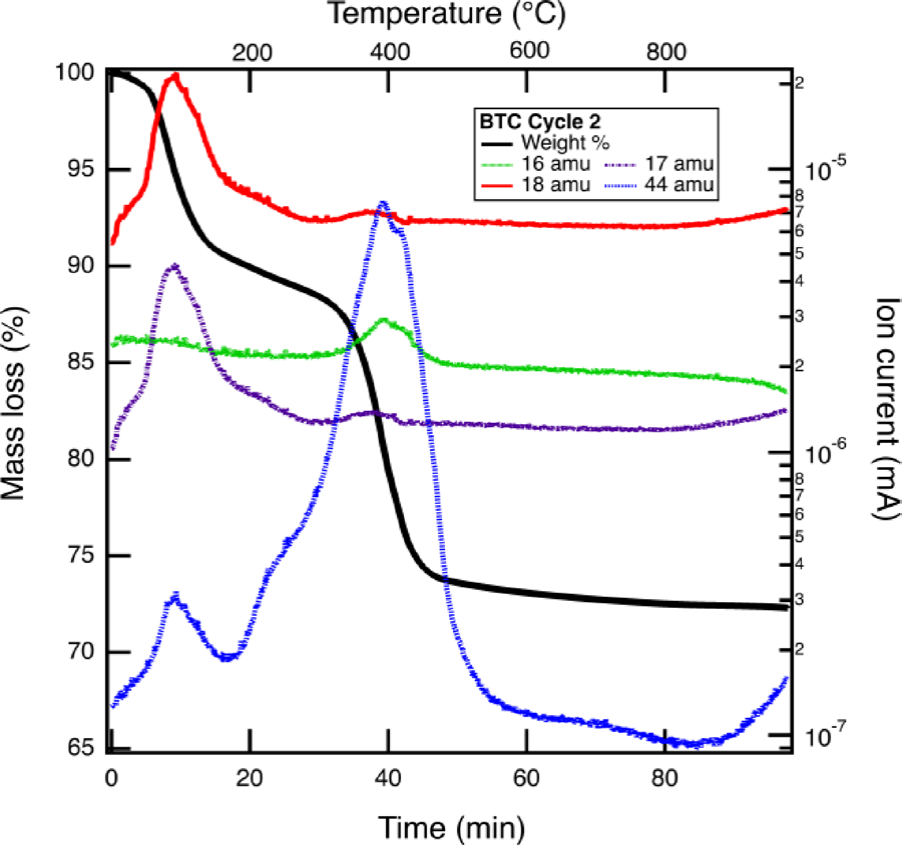
Representative TGA-MS data. Data shown here is from BTC carbonated at 100% relative humidity for 14 days in a second carbonation cycle. The mass loss for the TGA is shown in solid, black line, while the masses of some representative species are shown using colored lines and labelled according to their assignment. Mass 16 is oxygen, mass 17 is OH (likely an artifact of the MS process and not indicative of hydroxide in the sample), mass 18 likely corresponds to H_2_O, and mass 44 corresponds to CO_2_. TGA heating rate was 10 °C/minute.

Before testing the effect of repeated calcination/carbonation cycles on MgO reactivity, we characterized the effect of relative humidity on carbonation and analyzed the carbonation extent with thermogravimetric analysis with mass spectrometry (TGA-MS). A representative TGA-MS curve is shown in Fig. 1. From shape of the MS curve in Fig. 1 it is evident that the carbonation products are similar but the varying signal intensity of the MS signal corresponding to CO_2_ (44 atomic mass unit (AMU)) indicates different carbonation extent. The quantification of the carbonation efficiency of the MgO powders from TGA-MS mass loss alone is not straight-forward. This can be seen in Fig. 1, where there is a very broad peak for 18 AMU, which overlaps with the CO_2_ peak at 44 AMU in the region of 400 °C. This is due to the carbonation products containing water in their crystal structure (discussed below). This causes these two moieties to be liberated at overlapping temperatures and therefore the carbonation efficiency cannot be easily calculated without additional standardization since at different conditions, different carbonation products are formed. The mass loss can be used, however, to compare the relative carbonation efficiency if the carbonation products are similar composition. Above ~ 550 °C, minimal mass loss is observed, indicating the MgO has been regenerated. We also characterized a suite of reference materials using TGA-MS (see Figure S12 to S15), namely hydromagnesite (Mg_5_(CO_3_)_4_(OH)_2_·4H_2_O)^19^, magnesite (MgCO_3_), nesquehonite (MgCO_3_·3H_2_O)^20^ and artinite (Mg_2_(CO_3_)(OH)_2_·3H_2_O)^21^. However, we find that TGA-MS alone cannot be used to identify mineral phases after MgO carbonation and therefore employed X-ray diffraction for phase identification of carbonated MgO samples.

**Fig. 2:**
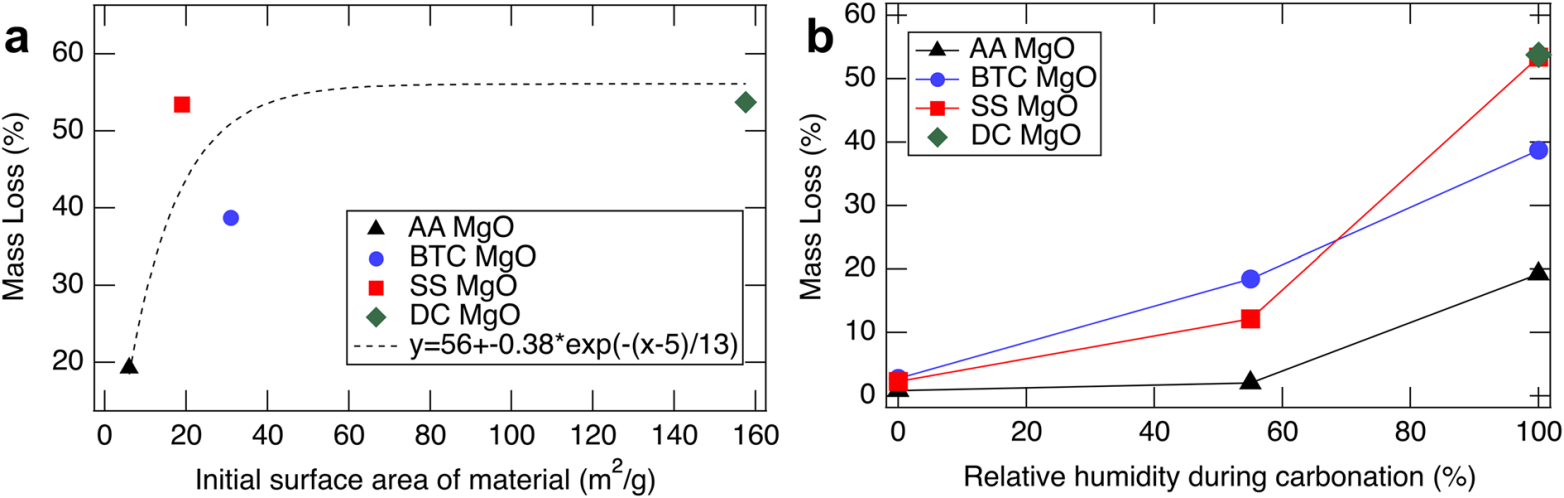
Mass losses of MgO powder heated to 700 °C as a function of (**a**) starting surface area of the pre-treated material at constant relative humidity (100%), and (**b**) relative humidity. The dashed black curve in a) is not predictive, but a fit intended to serve as a guide for the eye. All experiments shown in this figure were conducted at 25 °C and 1 bar CO_2_ for a duration of 14 days. Mass loss increases to ~ 55%, upon which the material is completely reacted, whereas increasing relative humidity is beneficial.

As an initial test to understand the carbonation efficiency of the different powders and the influence of different relative humidities on the carbonation efficiency, the mass loss as a function of the initial surface area and relative humidity are plotted in Fig. 2. Maximum carbonation extent was measured by sampling the top of each experimental vial because previous experiments had shown a reaction gradient as function of distance from the interface with the gas/vapor phase. In addition to the three commercial powders, a synthesized MgO powder with a comparably high surface area (~ 5x higher than the BTC) was also used (DC MgO). Due to practical limitations on the amount that could be synthesized, this material was not used for further tests beyond those in Fig. 2. Increasing mass loss roughly correlated with increasing surface area until the mass loss reaches ~ 55%, upon which no further mass loss is observed. Increasing relative humidity increases the mass loss. Qualitative analysis of the MS signals suggests that water content in the samples does increase, but so also does the CO_2_ content. Thus, in subsequent testing, 100% R.H. was used. After the initial tests presented in Figs. 1 and 2, samples were homogenized prior to analysis with the TGA-MS to better represent the average carbonation extent over the sample and increase reproducibility.

**Fig. 3:**
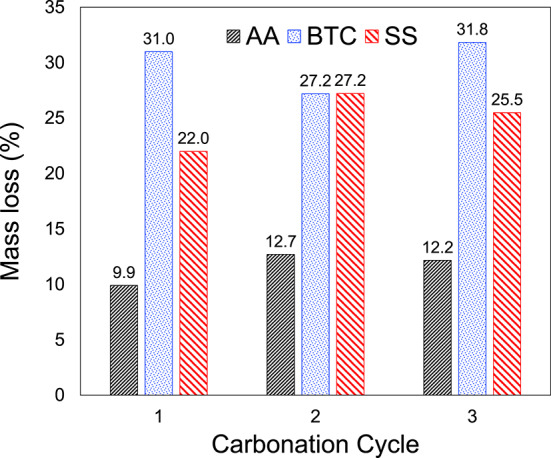
Mass loss at 700 °C measured by TGA-MS for three MgO powders (AA MgO, BTC MgO, SS MgO) in three calcination cycles.

The mass loss for Alfa Aesar (AA) powder after three different carbonation cycles (Fig. 3) did not vary significantly at an average of ~ 11.6% (first cycle: 9.9%, second cycle: 12.7%, and third cycle: 12.2%). The other two powders showed similar trends with unchanged mass loss for repeated carbonation cycles. The Beantown Chemical (BTC) powder showed an average mass loss for the three cycles of ~ 30% (first cycle: 31%, second cycle: 27.2%, and third cycle 31.8%), and Skysprings (SS) powder showed an average mass loss of ~ 24.9% (first cycle: 22.1%, second cycle: 27.1%, and third cycle: 25.5%). In terms of mass loss, no effect of calcination on the carbonation efficiency was observed, since the mass loss is very similar after the three carbonation cycles for all materials.

**Fig. 4:**
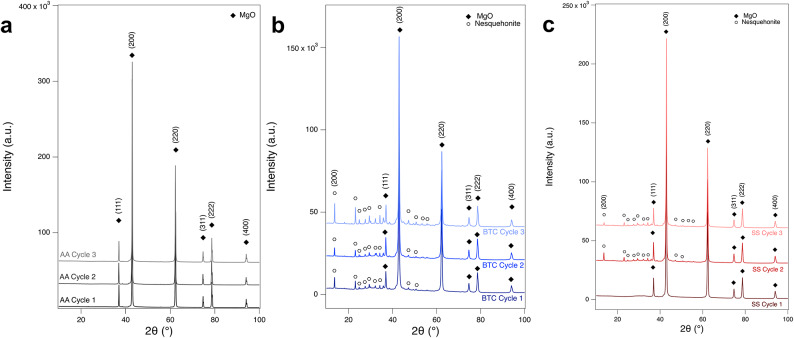
XRD of MgO powders after carbonation cycles. (**a**) AA MgO, (**b**) BTC MgO and (**c**) SS MgO.

XRD analysis with Rietveld refinement was performed to determine which crystalline carbonation products were formed. The XRD patterns after each carbonation cycle are shown in Fig. 4. Nesquehonite (MgCO_3_ ⋅ 3H_2_O) was the only hydrated carbonate observed after carbonation. The only other detected phase in the refinements was MgO.

**Fig. 5:**
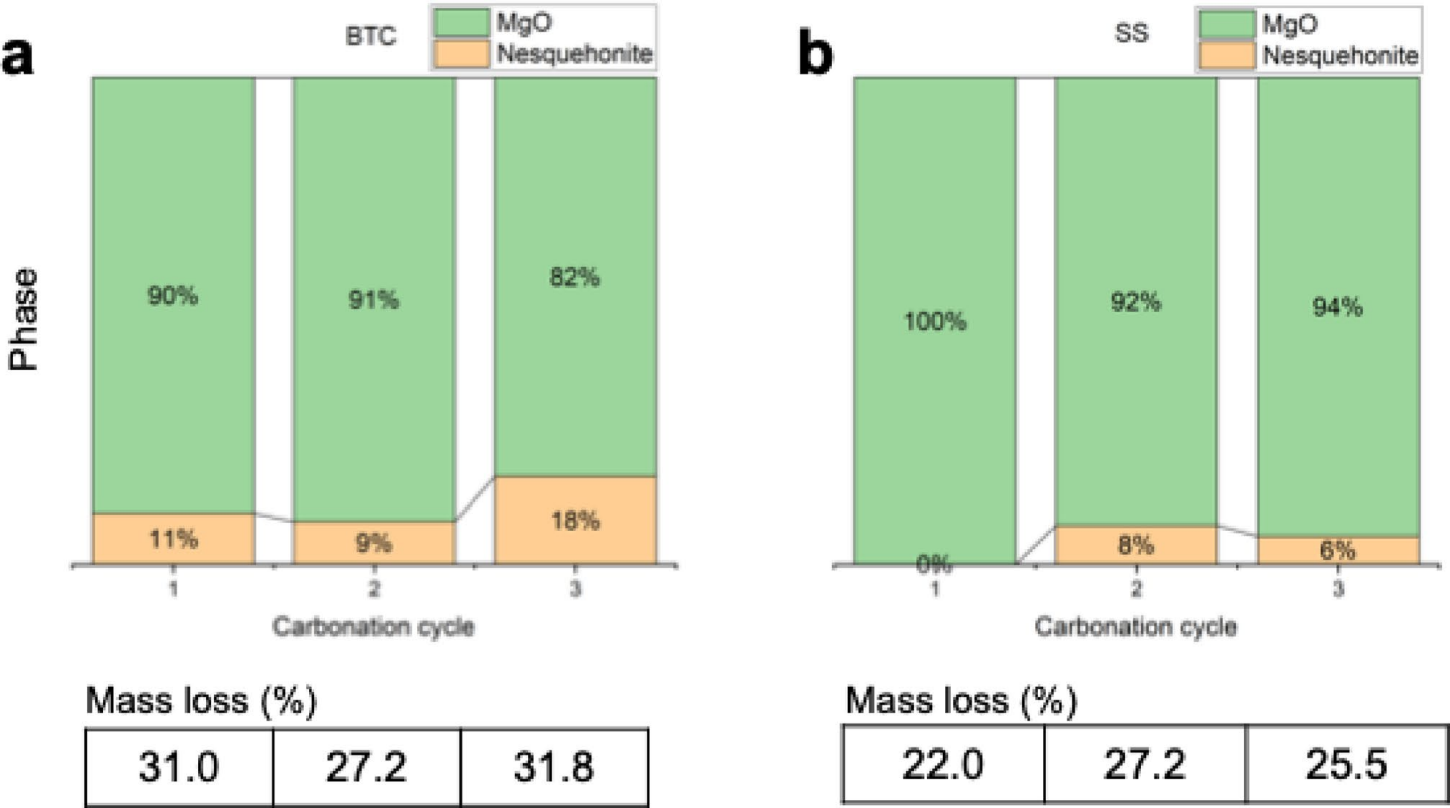
Phase distribution in (**a**) BTC and (**b**) SS MgO powders after carbonation as determined by Rietveld refinement.

Rietveld refinement of the nesquehonite content was performed to identify changes in the proportion of the crystalline products with calcining cycles and between the different MgO powders (Fig. 5). After the first carbonation cycle, the BTC samples contained 10.5 wt% nesquehonite. This slightly decreased in the second carbonation cycle to 8.8 wt% and then increased to 18.1 wt% in the third carbonation cycle. The weight% of nesquehonite in the carbonated SS MgO was lower than that in BTC. No nesquehonite was detected after the first carbonation cycle, 8 wt% after the second carbonation cycle and 5.8 wt% after the third carbonation cycle. No quantifiable amount of nesquehonite was present in the carbonated AA sample, although a trace was detected after the first carbonation. This suggests that the carbonation products were partially amorphous as mass loss was observed in the TGA data. Similar formation of amorphous Mg-carbonates has previously been observed^22–24^.

**Fig. 6:**
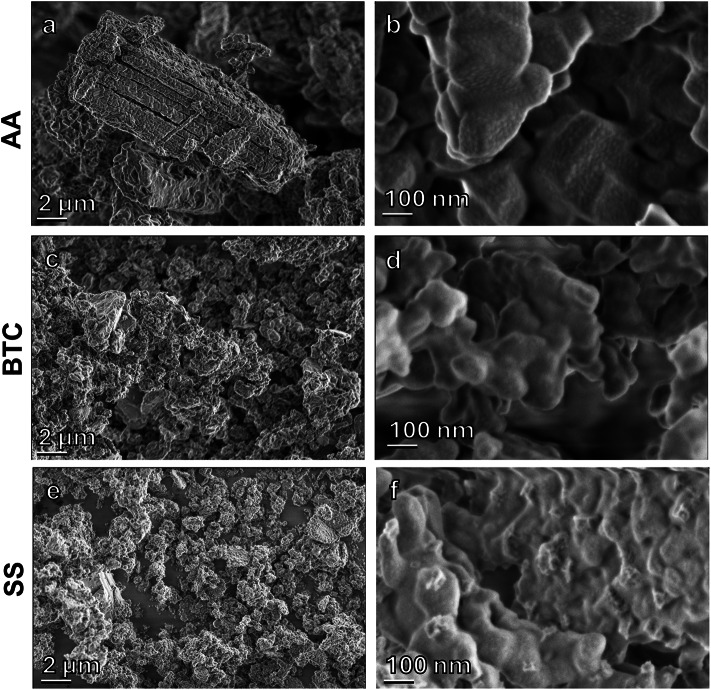
SEM imaging of MgO powders after carbonation (1st cycle). **a**) AA MgO showing formation of rods and formation of a rough surface layer on MgO cubes (**b**). **c**) BTC MgO showing aggregation of particles by a surface coating (**d**). **e**) SS MgO powder with a surface coating (**f**) with rough surface visible.

SEM imaging of the MgO powders after carbonation (Fig. 6) showed large rods (~ 10 μm) formed from the AA powder, which at higher magnification are visible to consist of individual MgO cubes. This nanocube alignment has previously been attributed to the hydroxylation reaction^13^ that can be expected to occur here as the experiments were conducted at 100% R.H. However, the starting product also showed this alignment. In addition, the surface of the AA cubes was covered with small bumps. For BTC and SS, similar particle surfaces and intergrowth were also observed, but the original particles were less distinct.

**Fig. 7:**
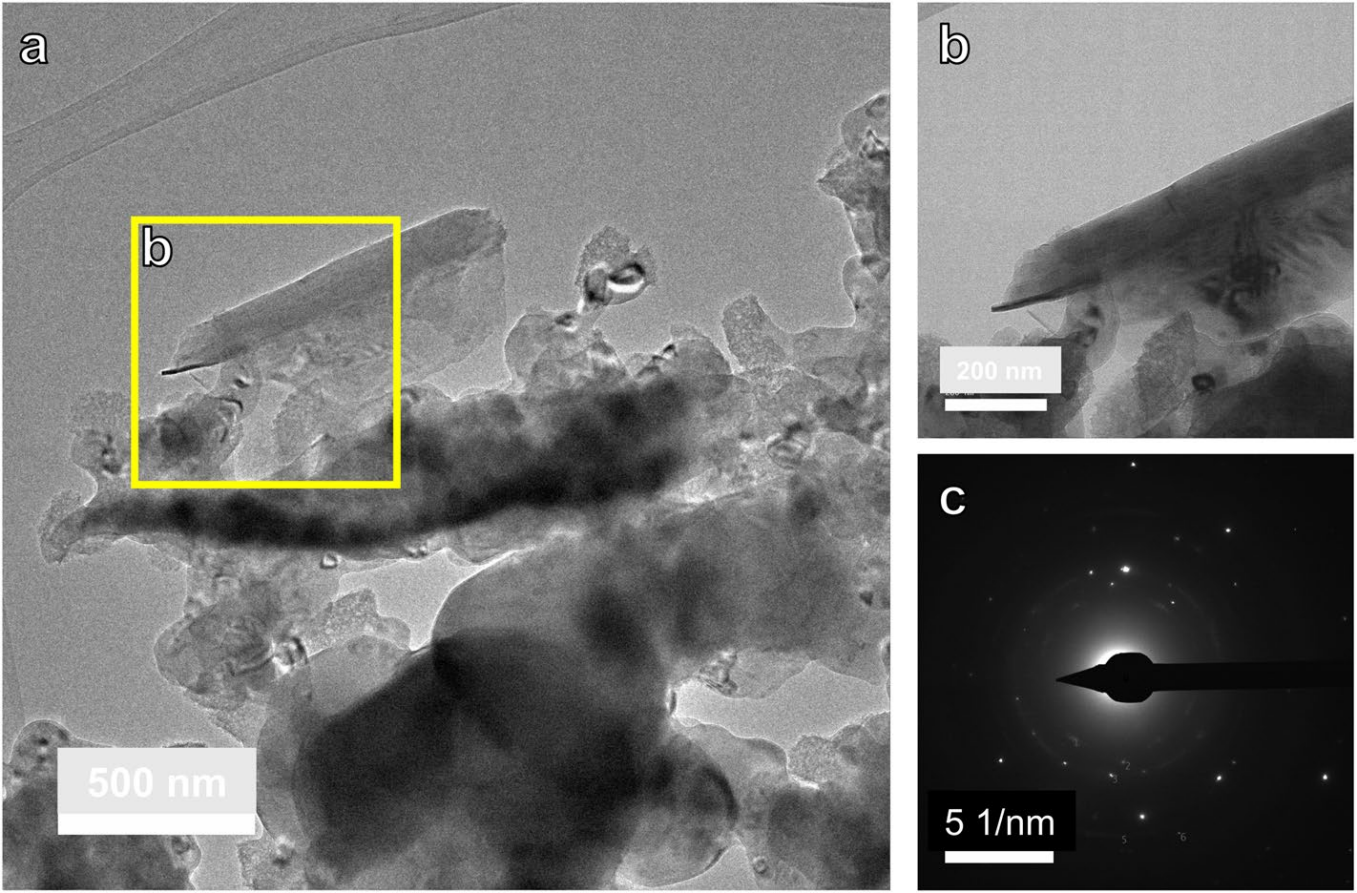
TEM characterization of the 3rd cycle reaction products of SS. (**a**) BF-TEM image of SS showing needle shaped reaction products. (**b**) A higher magnification image of the area highlighted in **a**). (**c**) selected area electron diffraction image of needle. Diffraction peaks match nesquehonite and underlying MgO.

Transmission electron microscopy bright field (TEM-BF) imaging of the 3rd cycle carbonation products of SS shows needle-shaped crystals of ~ 700 nm long (Fig. 7). The selected area electron diffraction (SAED) pattern showed that the needle-shaped crystal is nesquehonite, although underlying MgO reflections were also detected. This matches the Rietveld refinement data which showed that the reaction products contained 8 wt% nesquehonite.

### Effect of calcination on surface area and MgO crystallinity

**Fig. 8:**
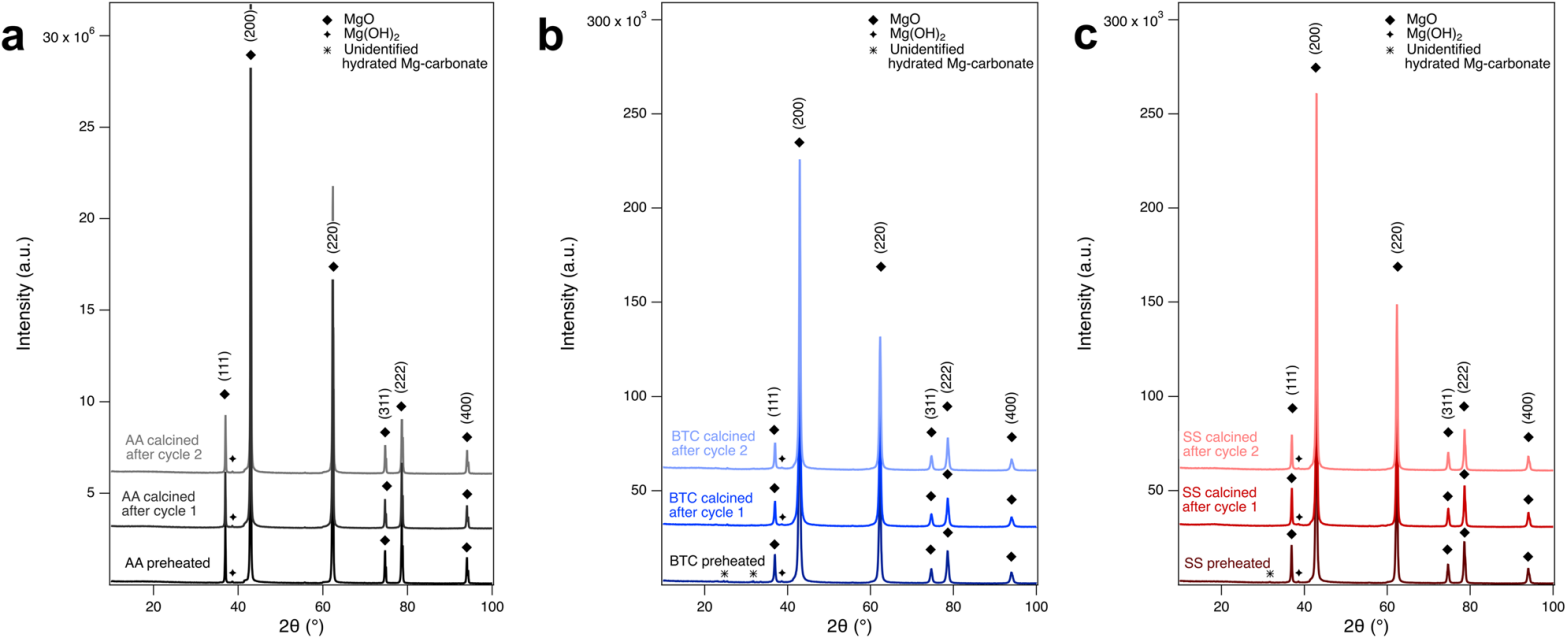
XRD patterns of the pre-heated starting materials and calcined products after one and two carbonation cycles. (**a**) AA, (**b**) BTC, and (**c**) SS. The star marks peaks at ~ 31°, likely hydrated Mg-carbonates that are present both in BTC and SS but are removed after the first cycle.

**Fig. 9:**
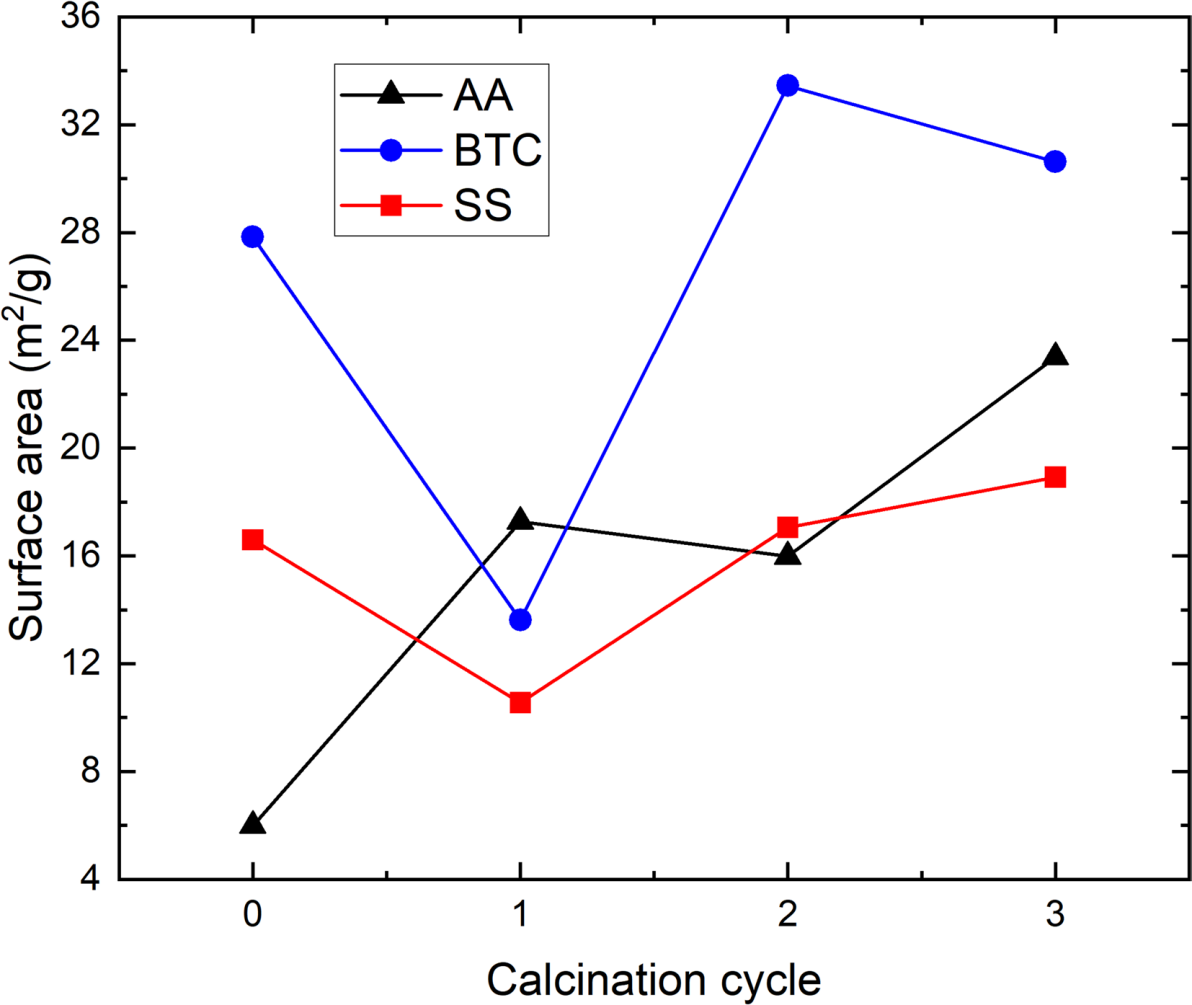
Surface area measurements (m^2^/g) from BET after calcination.

XRD characterization was conducted to test the effect of repeated calcination on MgO crystallinity and to determine whether all the carbonation products were removed during calcination. The XRD patterns of the pre-heated starting materials and the calcined ones after two cycles are practically identical (Fig. 8). Thus, the calcining process was successful at decomposing all the carbonation products and regenerating the MgO. All starting materials contained small amounts of brucite (Mg(OH)_2_) as evident by the peak at 38.5°, which is in reasonable agreement with the brucite (101) peak^25^ at 38.2° (Fig. 9). This might be due to rapid reaction of MgO with relative humidity forming Mg(OH)_2_. The pre-heated SS material contained an additional unidentified peak (marked with star in Fig. 9c) at 31.6°, which could possibly stem from a non-complete decomposition of hydrated carbonates, e.g., artinite^21^ has a peak at 31.98° and hydromagnesite^19^ at 31.5°. This peak at 31.6° is removed after the first calcination cycle (star in Fig. 8b + c). The pre-heated BTC material showed a similar peak at 31.7°, which likely also can be attributed to hydrated carbonates and is removed after the first calcination cycle.

**Fig. 10:**
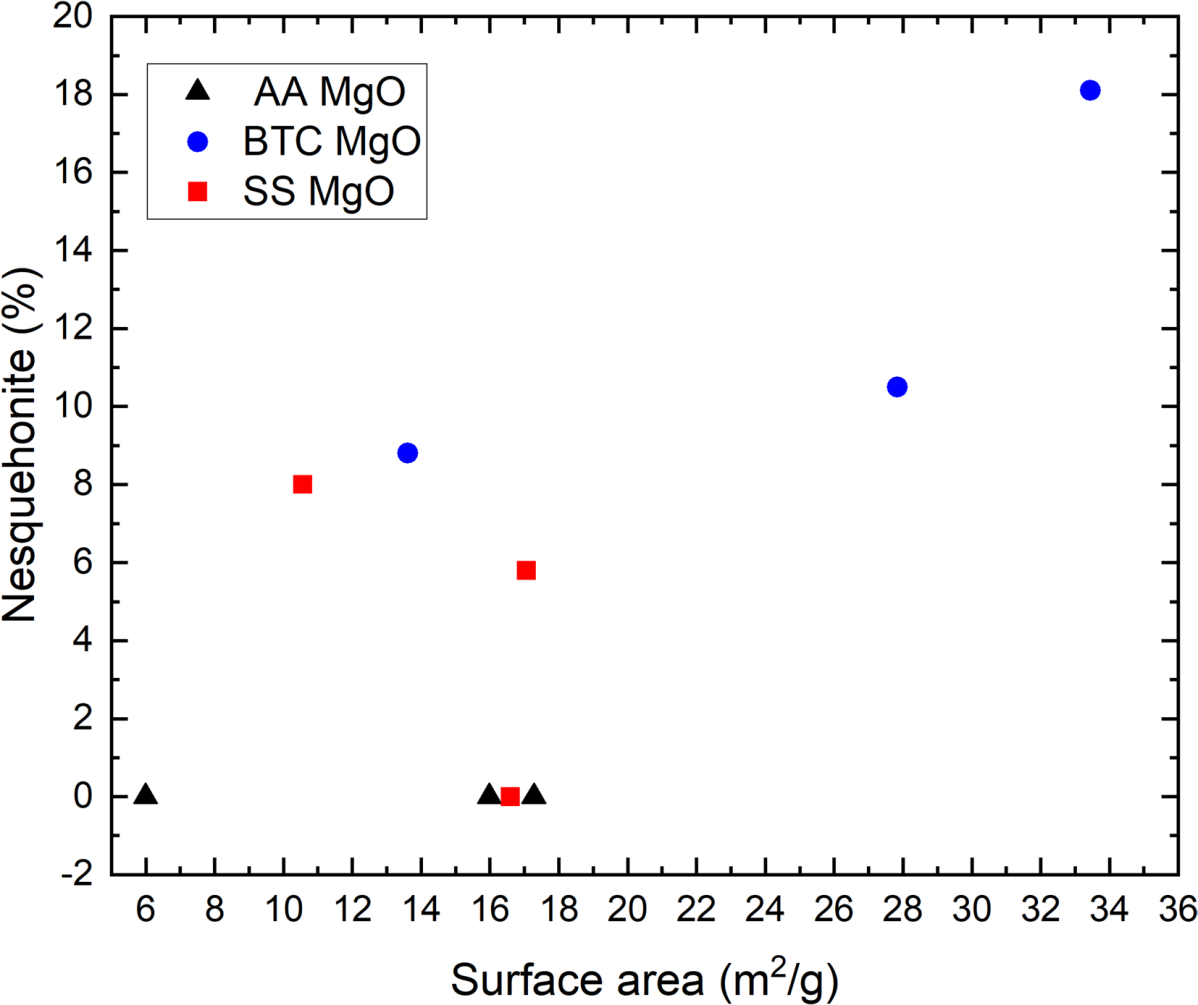
Weight percent nesquehonite (%) as a function of surface area (m^2^/g) for three MgO powders.

Changes in the BET surface area of the materials after the calcination cycles are shown Fig. 9. The AA MgO shows an increase in surface area from as received to the first cycle, likely due to the rapid release of water from hydrated phases that had formed during carbonation. For BTC and SS, the surface area dropped after the first calcination indicating possible intergrowth of particles. Overall, all three powders showed an increase of surface area after the third calcination cycle. No large decrease in surface area that would deleteriously affect future carbonation was observed that could be attributed to sintering. The differences between AA and BTC/SS may reflect the relative stabilities of the different nanocrystal shapes in the original starting material (Figs. 6 and 10).

## Discussion

**Fig. 11:**
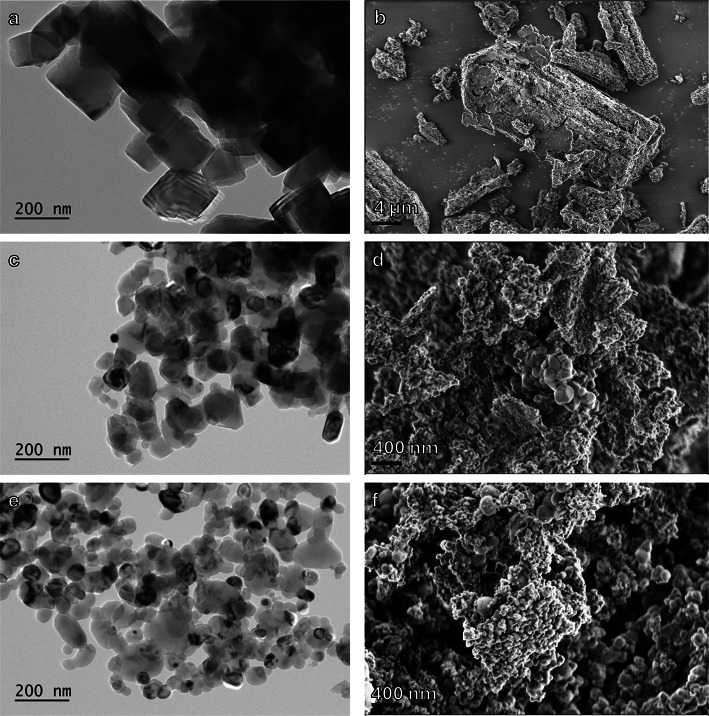
MgO nanopowder characterization prior to experiments using microscopy. (**a**) Bright Field Transmission Electron Microscopy (BF-TEM) image of AA, (**b**) Secondary electron scanning electron microscopy (SE-SEM) image of AA. (**c**) BF-TEM image of BTC, (**d**) SE-SEM image of BTC, (**e**) BF-TEM image of SS, (**f**) SE-SEM image of SS.

The extent of carbonation as determined from the TGA-MS mass loss clearly differs between the three materials and appears to be a function of their initial surface area or crystal form (Fig. 11). Carbonation progressed further for the BTC MgO (SS was similar), and was weakest for the AA MgO, the initial powders with the highest and lowest initial surface area (27.8 and 6.0 m^2^/g). Comparing the mass to the changing surface area (Fig. 11) shows that the greater the surface area the greater the mass loss. There is only one datapoint for SS (cycle 1) that shows a greater mass loss despite a decrease in surface area. This reflects the greater amount of nesquehonite that formed in the SS product after the second carbonation cycles. However, the morphology of the AA MgO consists only of (100) facets, which have been shown to be less stable than (111) and (110)^26^, which forms approximately the shapes of BTC and SS, although they are clearly not well-formed octahedra or dodecahedra.

While some nesquehonite is observed in the samples using XRD (Fig. 4), the maximum mass loss would represent complete conversion if the products matched the composition of hydromagnesite ((MgCO_3_)_4_Mg(OH)_2_∙4H_2_O), which would be a 53% mass loss. If the reaction products matched the composition of nesquehonite (MgCO_3_∙(3H_2_O)), complete conversion would be 71% mass loss, and that of magnesite (MgCO_3_) would be 50% mass loss. However, the latter is clearly not the case since the water MS signals from 18 AMU and 17 AMU are significant.

**Fig. 12:**
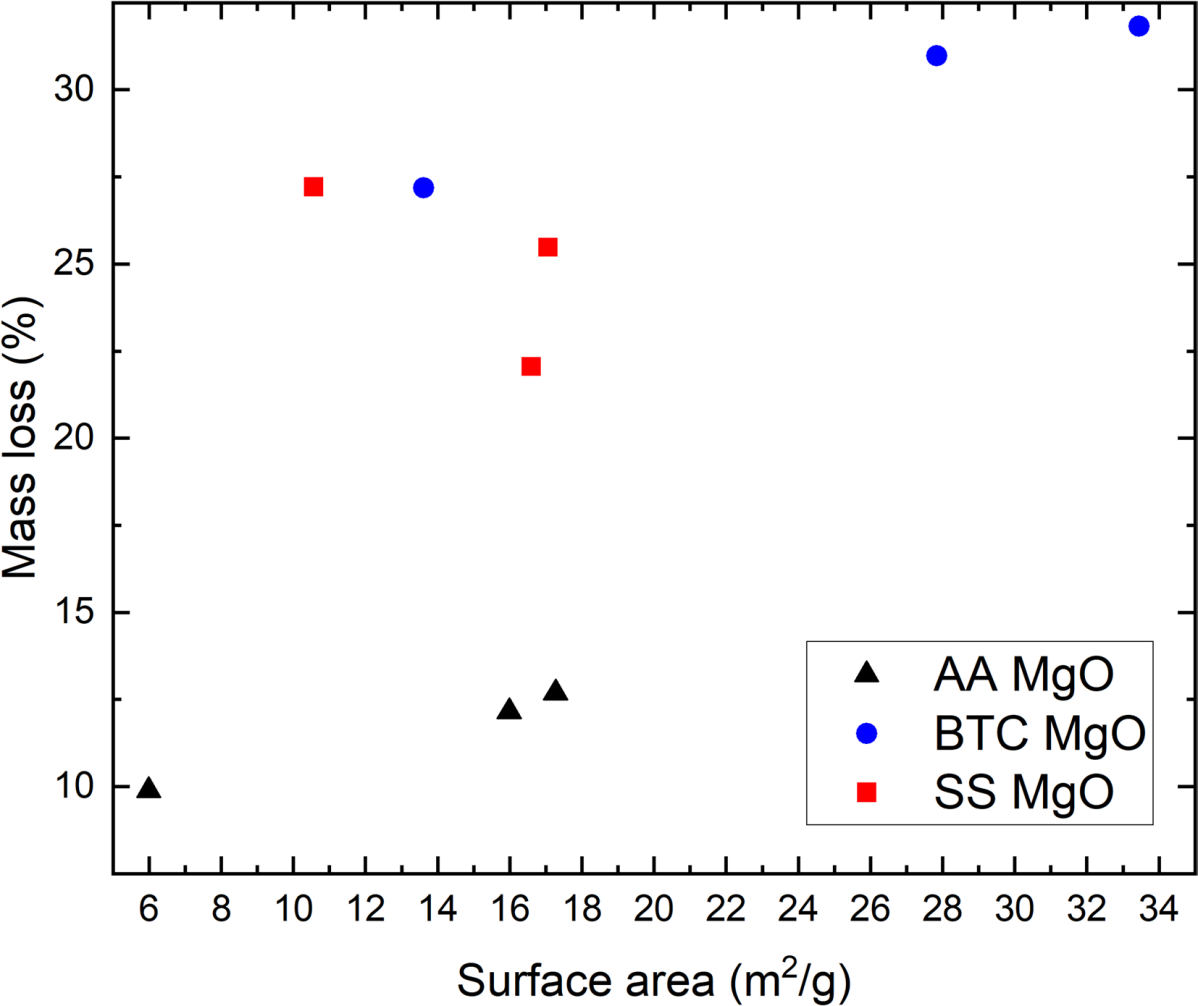
Mass loss (%) measured by TGA-MS as a function of BET surface area (m^2^/g). Mass loss was calculated by subtracting the relative change in mass (%) at 700 °C from 100.

Comparing the TGA-MS mass loss to the weight% of nesquehonite formed as determined via Rietveld refinement of XRD data (Fig. 12), strongly suggest that not all the carbonation products can be crystalline. For example, XRD of SS after the first carbonation cycles shows no evidence of nesquehonite formation but TGA-MS showed mass loss of ~ 22% and the presence of CO_2_ in the MS spectrum. This indicates that amorphous carbonates have formed, which is further supported by the broad hump in the XRD diffractogram around 30° (Fig. 4). The same result was observed for the AA MgO after all cycles. No quantifiable amount of nesquehonite was found, but the average weight loss of 11.6%, CO_2_ was clearly present in the MS data, and a hump was again observed in the XRD pattern around 30° (Fig. 4). In the case of BTC, the TGA-MS mass loss was essentially constant at ~ 30%. However, the nesquehonite content increases with each carbonation cycle. The first and second carbonation cycle yielded 10.5 and 8.8 wt% nesquehonite, respectively, but the third generated 18.1 wt% nesquehonite. This indicates that increased cycling increased the crystallinity of the carbonation product. The observation of amorphous materials is consistent with observations in other studies on MgO carbonation^15,22^. SEM imaging of MgO powders after carbonation shows the smoothing of the surface as well as intergrowth of particles, which indicates that a surface layer might have formed (Fig. 6), which could be composed of this amorphous material.

### Implications

In conclusion, we observed a strong correlation between the carbonation efficiency of MgO powders and their surface area. This shows that it will be more beneficial to utilize a MgO powder with higher surface area for direct air capture, as would be expected. Lower surface area MgO powder (AA) shows around 10% mass loss after carbonation in presence of 100% R.H. for two weeks, whereas the higher surface area MgO powders (BTC, SS) showed 22–32% mass loss. However, carbonation efficiency also correlated with at least the initial morphology of the MgO crystals. Here, the AA MgO morphology consisting of (100) facets seem to be less beneficial for carbonation compared to the BTC/SS MgO powders of octahedra or dodecahedra morphology. Therefore, surface area and morphology might either individually or jointly control the reaction rate.

Our results also showed that carbonation/calcination did not lead to a reduction of surface area or reactivity as measured by mass loss in TGA-MS, at least over three mineral looping cycles. This contrasts with previous studies of mineral looping at elevated temperatures^11,12,14,17^ in which a reduction of surface area was observed within the first three cycles. We hypothesize that this is mainly due to differences in reaction mechanism at ambient temperatures in the presence of relative humidity. Previous studies postulated that MgO carbonation in presence of humidity initially proceeds via formation of brucite (Mg(OH)_2_), which then dissolves and forms a carbonate. Since the molar volume (V_m_) of brucite (V_m_ = 24.63 cm^3^/mol)^27^ is larger than that of MgO (V_m_ = 11.248 cm^3^/mol)^27^, this volume-increasing reaction can lead to fracturing of the material^28,29^. We find that brucite is present in our MgO powders even immediately after calcining, likely due to reaction with water present as relative humidity in the air. We then observe the formation of an amorphous carbonate phase as well as hydrated Mg-carbonate in the form of nesquehonite. Overall, our results confirm that MgO is a promising sorbent material for CO_2_ capture at low temperatures in presence of humidity and can suggest it be used in a looped process without significant calcination-induced surface area loss. It further suggests that the mechanisms involved in metal oxide looping at elevated temperatures may be radically different from those controlling the hydration/carbonation processes at the relatively low temperatures needed for ambient carbon capture.

## Methods

### Materials

Three high-purity MgO powders were used for carbonation/calcination cycles (Fig. 10). The nanopowders were sourced from Alfa Aesar (AA, 325 mesh, 99.95 wt% purity), Beantown chemicals (BTC, 40–60 nm, 99 wt% purity), and Skysprings Nanomaterials Inc. (SS, 20 nm, 99 + wt% purity). The specific surface area of the as received starting materials was measured via BET analysis (method description see below) at 5, 16, 32 m^2^/g for AA, SS, and BTC respectively. Each set of materials were pre-treated by heating overnight at 400 °C to provide a uniformly treated material since each of the received powders was of unknown age and carbonation by ambient air can be expected. The temperature was increased over a period of 2 hours to prevent sudden changes in the material. The surface areas of the heated materials were 6, 17, 28 m^2^/g for AA, SS, and BTC respectively. These surface areas are close to the surface areas of the as received materials and suggests only minor, if any, changes to the materials due to the pre-treatment. SEM and TEM examination of the pre-treated powders showed that the AA powder was composed of nanocubes arranged in larger rods (Fig. 10b, d, e). BTC and SS powders were composed of agglomerated rounded nanoparticles with observable size differences, especially in the case of the SS powder (Fig. 10).

High surface area Nano-MgO (referred here as DC MgO) was synthesized by calcinating nano-brucite (Mg(OH)_2_) synthesized according to previous protocols^30^. To be specific, 6 g of NaOH pellets were dissolved in a solution containing 70 mL of 1:1 ethanol and deionized water with a magnetic stir bar at 700 rpm. After NaOH pallets were fully dissolved, 3.446 g of solid Mg(NO_3_)_2_∙6H_2_O were directly placed in the stirred NaOH solution. This solution was stirred for ~ 3 h. After that, the stirring was turned off and precipitates were left to settle overnight. The clean supernatant was decanted, and precipitates were evenly dispensed to six 50 mL centrifuge tubes. They were centrifuged at 12,000 rpm for 30 min four times at room temperature. After each centrifugation cycle, the supernatant was decanted, and deionized water was added to almost fill the tubes (~ 40 mL) to remove residual salts and base. The precipitates were consolidated to reduce the number of centrifuge tubes. The precipitates were sonicated for 30 min at room temperature three times. After the fourth cycle, the supernatant was decanted, and the precipitate was dried in a vacuum oven at ~-19 psi relative to room pressure and ~ 75℃. After drying for five days, the precipitates were gently ground using an agate mortar. After that, the dry powder was placed in a crucible and calcined in an oven at 500 °C for 3 h. XRD and SEM images are given in the supporting information. Surface area was determined using BET measurements (details given below) and were 158 m²/g.

Reference TGA-MS for hydrated Mg-carbonate phases was collected using a TA Instruments TGA 5500 coupled with a Discovery quadrupole mass spectrometer (MS). Samples were measured at a heating rate of 10 °C/minute to a temperature of 1000 °C under an argon flow. Since mass loss between 700 °C and 1000 °C was minimal (< 1%), mass loss at 700 °C was used to evaluate carbonation efficiency. Hydromagnesite was purchased from Baker. Nesquehonite was synthesized by reacting MgO with water and elevated CO_2_ for prolonged times according to published protocols^31,32^. Artinite and magnesite were purchased at the Tucson Gem and Mineral Show.

### Carbonation and calcination loops

Three fourteen-day carbonation/calcination loops at 25 °C were performed on each material using a custom-built, stainless steel multi-vessel system^33^ at 100% relative humidity. Glass vials containing the MgO powders (~ 0.2 g) were placed in a Teflon-lined reaction vessel, which was then connected to a CO_2_ tank at 1 bar CO_2_ above atmospheric pressure (gauge). To maintain a constant relative humidity, approximately 2 mL of deionized water was placed at the bottom of the reaction cell, around the glass vials but not in direct contact with MgO. For experiments at 55% relative humidity, saturated potassium chloride solution was added instead of deionized water. For experiments in absence of humidity, no liquid was added into the reaction vessel.

After the end of the carbonation experiments, samples were dried under a N_2_ atmosphere at 70 °C overnight. The N_2_ atmosphere was provided by evacuating and backfilling the oven with gas three times. Calcination after each carbonation cycles was done in small temperature increments to avoid violent decomposition of the hydrates. The temperature rates and steps during calcination were chosen with guidance from the TGA-MS by holding at the average temperature of the weight loss steps in the TGA-MS spectra, followed by a final hold at 500 °C to ensure all hydrates were decomposed. The ramp rate for calcination was 2 °C/min with 30-minute holds at 115 °C, 400 °C, and 500 °C.

Initial measurements of the carbonation extent were performed by taking samples from the top of each vial. This represents the maximum carbonation extent. However, a reaction gradient from the top to the bottom of each vial was observed and therefore, later measurements of the carbonation extent were done using homogenized contents of each vial.

### Thermogravimetric analysis and mass spectrometry

The extent of carbonation in each sample was analyzed by thermogravimetric- mass spectrometric analysis (TGA-MS) using a TA Instruments TGA 5500 coupled with a Discovery quadrupole mass spectrometer (MS). Samples were measured at a heating rate of 10 °C/minute to a temperature of 1000 °C under an argon flow. Since mass loss between 700 °C and 1000 °C was minimal (< 1%), mass loss at 700 °C was used to evaluate carbonation efficiency.

### Scanning and transmission electron microscopy characterization

Scanning electron microscopy was performed using a Merlin FE-SEM (Zeiss) microscope operated at 10 kV. A small part of the sample was deposited on a carbon sticker with no additional coating. Images were taken at magnifications from 5k× to 100k× by mixing signals from the SE2 and the in-lens detectors at equal percentages. Transmission electron microscopy of the starting materials was done using a JEOL JEM-2100 F TEM/STEM 200 kV field-emission system at magnifications up to 25k×. Bright field images and diffraction patterns of the carbonated materials were performed using a FEI Titan (60–300 kV) aberration-corrected STEM operating at 300 kV. Samples were prepared by sonicating a small amount of the powder in isopropanol for 5–10 min and depositing a drop of the suspension over a Cu grid with lacey carbon or plain carbon film.

### XRD characterization

X-ray diffraction patterns were collected using a Bruker D2 PHASER benchtop diffractometer that operated at 30 kV and 10 mA with a Cu k_α_ source (λ = 1.54056 Å). Data were acquired over a 2θ range from 10 to 100°, with a step size of 0.03°, and a 3 s time per step. Rietveld refinement was performed using HighScore Plus (Panalytical) and ICDD references 04-004-8990 and 01-079-9947 for MgO and nesquehonite, respectively.

XRD characterization of standards for TGA-MS analysis (artinite, nesquehonite and hydromagnesite) were obtained using synchrotron XRD at the GeoSoilsEnviroCARS 13-BMC beamline at the Advanced Photon Source at the Argonne National Laboratory. Samples were powdered and mounted in Kapton capillaries (Cole-Parmer, 0.0577’’ inner diameter, 0.615’’ outer diameter). The monochromatic wavelength was 0.8204 Å as determined using LaB_6_ standard. Sample-to-detector distance was 100 mm and a 300 × 150 μm window of the X-ray beam was focused on the center of the capillary. Preferred orientation effects were reduced by rotating the samples through a 30° phi angle along the long axis of the capillary during the measurement. Diffraction rings were collected using a Dectris PILATUS 1 M pixel array detector, which were integrated using DIOPTAS^34^. Detection limit for synchrotron XRD is ~ 1 wt%.

### BET surface area

The surface area of the samples was measured using a Micromeritics 3Flex Brunauer-Emmett-Teller (BET) instrument. The samples were degassed in a Smartvac system at 150 °C for 12 h. The surface area of the degassed samples was measured using N_2_ as the adsorbate and liquid nitrogen as the cryogen. Eleven pressure points were measured, and the surface area was calculated using the standard BET Eq^35^.

## Supplementary Information

Below is the link to the electronic supplementary material.

Supplementary Material 1

## Data Availability

Associated research data is available from the corresponding author upon request.
