# Correction: Effect of multiple calcination cycles on CO_2_ capture efficiency during carbonation of MgO in a mineral looping process

**DOI:** 10.1038/s41598-026-38026-4

**Published:** 2026-02-02

**Authors:** Elena Tajuelo Rodriguez, Lawrence M. Anovitz, Sai Adapa, Ke Yuan, Dale Hensley, Dong Youn Chung, Matthew G. Boebinger, Andrew G. Stack, Juliane Weber

**Affiliations:** 1 Nuclear Energy and Fuel Cycle Division, Oak Ridge National Laboratory, Oak Ridge, 37831 USA; 2 Chemical Science Division, Oak Ridge National Laboratory, Oak Ridge, 37831 USA; 3 Center for Nanophase Materials Sciences, Oak Ridge National Laboratory, Oak Ridge, 37831 USA; 4 Department of Geosciences, The Pennsylvania State University, State College, PA 16802 USA

Correction to: *Scientific Reports* 10.1038/s41598-025-23708-2, published online 10 November 2025

The original version of this Article contained errors in the Figure legends of Figure 10, 11 and 12. The legends of these Figures were inadvertently switched.

The legend of Figure 10:

“MgO nanopowder characterization prior to experiments using microscopy. (**a**) Bright Field Transmission Electron Microscopy (BF-TEM) image of AA, (**b**) Secondary electron scanning electron microscopy (SE-SEM) image of AA. (**c**) BF-TEM image of BTC, (**d**) SE-SEM image of BTC, (**e**) BF-TEM image of SS, (**f**) SE-SEM image of SS.”

now reads:

“Weight percent nesquehonite (%) as a function of surface area (m^2^/g) for three MgO powders.”

The legend of Figure 11:

“Mass loss (%) measured by TGA-MS as a function of BET surface area (m^2^/g). Mass loss was calculated by subtracting the relative change in mass (%) at 700 °C from 100.”

now reads:

“MgO nanopowder characterization prior to experiments using microscopy. (a) Bright Field Transmission Electron Microscopy (BF-TEM) image of AA, (b) Secondary electron scanning electron microscopy (SE-SEM) image of AA. (c) BF-TEM image of BTC, (d) SE-SEM image of BTC, (e) BF-TEM image of SS, (f) SE-SEM image of SS.”

The legend of Figure 12:

“Weight% nesquehonite (%) as a function of surface area (m^2^/g) for three MgO powders.”

now reads:

“Mass loss (%) measured by TGA-MS as a function of BET surface area (m2/g). Mass loss was calculated by subtracting the relative change in mass (%) at 700 °C from 100.”

The original Article has been corrected.

