# Supplementary material for: Effect of multiple calcination cycles on CO2 capture efficiency during carbonation of MgO in a mineral looping process
Source: Sci Rep. 2025 Nov 10;15:39193. doi: 10.1038/s41598-025-23708-2 (PMC12603058; doi:10.1038/s41598-025-23708-2)
Supplement: Supplementary file 1 — Supplementary Material 1 [file 41598_2025_23708_MOESM1_ESM.docx]

Supporting Information

For

Effect of Multiple Calcination Cycles on CO_2_ Capture Efficiency During Carbonation of MgO in a Mineral Looping Process

Elena Tajuelo Rodriguez^1^, Lawrence M. Anovitz^2^, Sai Adapa^2^, Ke Yuan^2^, Dale Hensley^3^, Dong Youn Chung^4^, Matthew G. Boebinger^3^, Andrew G. Stack^2^, Juliane Weber^2,*^

^1^Nuclear Energy and Fuel Cycle Division, Oak Ridge National Laboratory, Oak Ridge, 37831, USA

^2^Chemical Science Division, Oak Ridge National Laboratory, Oak Ridge, 37831, USA

^3^Center for Nanophase Materials Sciences, Oak Ridge National Laboratory, Oak Ridge, 37831 USA

^4^Department of Geosciences, The Pennsylvania State University, State College, PA, 16802, USA.

1. Table of Contents

[2.	Plots of Thermogravimetric Analysis with Mass Spectrometry (TGA-MS)	3](#_Toc209430742)

[3.	Characterization of nano MgO (DC MgO)	8](#_Toc209430743)

[4.	Reference Material Characterization	9](#_Toc209430744)

[5.	References	11](#_Toc209430745)

# 2. Plots of Thermogravimetric Analysis with Mass Spectrometry (TGA-MS)

Figure S 1	TGA-MS analysis of AA reacted for 1 carbonation cycle.

Figure S 2	TGA-MS analysis of AA reacted for 2 carbonation cycles.

Figure S 3	TGA-MS analysis of AA reacted for 3 carbonation cycles.

Figure S 4 TGA-MS analysis of BTC reacted for 1 carbonation cycle.

Figure S 5 TGA-MS analysis of BTC reacted for 2 carbonation cycles.

Figure S 6 TGA-MS analysis of BTC reacted for 3 carbonation cycles.

Figure S 7	TGA-MS analysis of SS reacted for 1 carbonation cycle.

Figure S 8	TGA-MS analysis of SS reacted for 2 carbonation cycles.

Figure S 9	TGA-MS analysis of SS reacted for 3 carbonation cycles.

# 3. Characterization of nano MgO (DC MgO)

Figure S 10	X-ray diffraction characterization of high surface area MgO (DC MgO) showing MgO peaks. All observed peaks are MgO peaks.

Figure S 11	Scanning electron microscopy characterization of DC MgO.

# 4. Reference Material Characterization

Figure S 12 Reference TGA-MS data for hydromagnesite (Mg_5_(CO_3_)4(OH)_2_·4H_2_O). a) XRD pattern of hydromagnesite compared to reference hydromagnesite data^1^. b) TGA-MS data for hydromagnesite.

Figure S 13 Reference TGA-MS data for nesquehonite (MgCO_3_·3H_2_O). a) XRD pattern of nesquehonite compared to reference nesquehonite data^2^. b) TGA-MS data for nesquehonite.

Figure S 14 Reference TGA-MS data for artinite (Mg_2_ (CO_3_)(OH)_2_·3H_2_O). a) XRD pattern of artinite compared to reference artinite data^3^. b) TGA-MS data for artinite.

Figure S 15 Reference TGA-MS data for magnesite (MgCO_3_). a) XRD pattern of artinite compared to reference magnesite data^4^. b) TGA-MS data for magnesite.

Figure S 16	Mass loss (%) measured by TGA-MS as a function of nesquehonite content (%) based on the XRD Rietveld refinement.

# 5. References

(1) Akao, M.; Iwai, S. The hydrogen bonding of hydromagnesite. *Structural Science* **1977**, *33* (4), 1273-1275.

(2) Stephan, G.; MacGillavry, C. H. The crystal structure of nesquehonite, MgCO3. 3H2O. *Structural Science* **1972**, *28* (4), 1031-1033.

(3) Akao, M.; Iwai, S. The hydrogen bonding of artinite. *Structural Science* **1977**, *33* (12), 3951-3953.

(4) Markgraf, S. A.; Reeder, R. J. High-temperature structure refinements of calcite and magnesite. *American Mineralogist* **1985**, *70* (5-6), 590-600.
